# Blood Cell Separation Using Polypropylene-Based Microfluidic Devices Based on Deterministic Lateral Displacement

**DOI:** 10.3390/mi14020238

**Published:** 2023-01-17

**Authors:** Koji Matsuura, Koji Takata

**Affiliations:** 1 Department of Bioscience, Faculty of Life Science, Okayama University of Science, Okayama 700-0005, Japan; 2 Life Materials Development Section, Human Life Technology Research Institute, Toyama Industrial Technology Research and Development Center, Nanto 939-1503, Japan

**Keywords:** deterministic lateral displacement, polypropylene-based microfluidic device, blood cell separation, microscopic analysis of blood cells

## Abstract

Mammalian blood cell separation methods contribute to improving the diagnosis and treatment of animal and human diseases. Microfluidic deterministic lateral displacement (DLD) devices can sort cells based on their particle diameter. We developed microfluidic DLD devices with poly(propylene)-based resin and used them to separate bovine and human red blood cells (RBCs) and white blood cells (WBCs) without electric devices. To determine the critical cut-off diameter (D_c_) of these devices, we used immunobeads with a diameter of 1–20 μm. The D_c_ values of the microfluidic DLD devices for the immunobeads in the experiments were similar to the calculated D_c_ values (8–10 μm). Results from bovine blood cell separation experiments suggest that lymphocytes and neutrophils can be separated from diluted, whole blood. Human RBCs were occasionally observed in the left outlet where larger particles with diameters closer to the D_c_ value were collected. Based on the D_c_ values, human neutrophils were sorted to the left outlet, whereas lymphocytes were observed in both outlets. Although microfluidic channel optimization is required for the concentration of sorted cells, the microfluidic DLD device prepared with a poly(propylene)-based resin has the potential for clinical use.

## 1. Introduction

**Figure 1:**
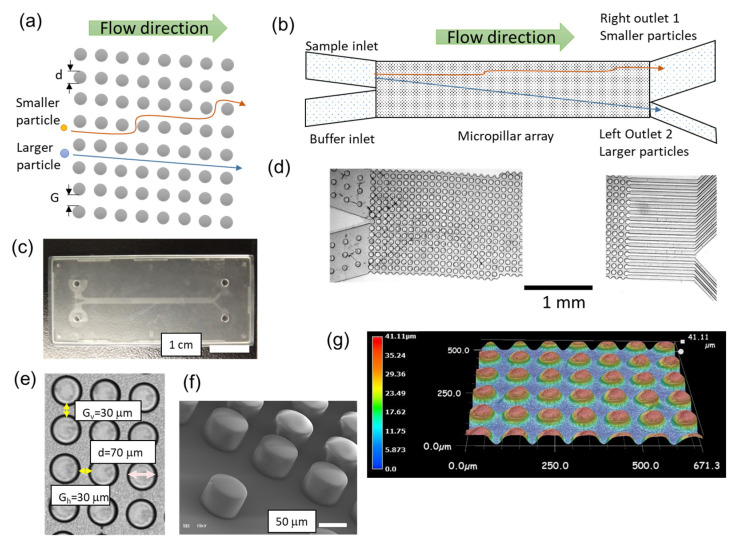
(**a**) Schematic of the deterministic lateral displacement (DLD) principle for particle separation. (**b**) Microfluidic channel arrangement in this study. (**c**) A photograph of the polypropylene (PP)-based microfluidic DLD device. The size of this chip is 25 mm × 58 mm. (**d**) Microscopic images of the inlets and outlets of the microfluidic DLD device (Chip 1). (**e**) An optical microscopy image showing the pillar size and gaps of the micropillars. (**f**) A SEM image of the micropillars. (**g**) A cross-section of the three-dimensional reconstructed image. A color contour shows the height of the microstructures. The heights of the pillars are approximately 40 μm.

Deterministic lateral displacement (DLD) devices have been used for the size-based separation of microspheres and mammalian cells, such as blood cells and bacteria [1,2,3,4,5,6,7,8,9,10,11,12,13,14,15,16,17,18,19,20,21,22,23]. Microfluidic DLD devices contain microfluidic channels with micropillar obstacles. A microsphere in the microfluidic DLD channel is acted on by lift force from the pillar and drag force to the microspheres (Figure 1a) [5]. The critical cut-off diameter (D_c_) for microparticles is dependent on the size of the gaps between the pillars (*g*) and the array rotation angle (θ), as shown in Equation (1), when the lateral gap and downstream gap are the same [4].

D_c_ = 1.4*g*(tanθ)^0.48^ (1)

As shown in Figure 1b, smaller particles remain within a flow stream, and large particles are displaced at each obstacle [2]. D_c_ depends on the pillar arrangement, pillar shape, and microparticle concentration [4,6]. A combination of microfluidic methodologies, including microfluidic DLD devices and digital process automation technologies, has been suggested for bioanalyses, such as antibody analysis of sorted cells and single-cell enzymatic secretion [3,17]. The advantages of microfluidic DLD devices over other microfluidic separation devices include a high separation efficiency and simple fluid control [5,6,7]. On the basis of lithography and microscale fabrication technologies, particle separation using DLD has been performed at a diameter scale of 1–50 μm, which offers the potential for biomedical applications involving cell separation [3,17,18,19].

Blood cell separation via microfluidic DLD devices has potential applications in clinical diagnosis and treatment [17,18,19]. Studies have proposed microfluidic DLD device applications such as automated diagnostic systems and chimeric antigen receptor T cell or other therapeutic cell-manufacturing processes [18]. The microfluidic DLD devices can provide the enrichment or depletion of common or rare cells, apheresis, and patient-specific immune signatures [17,19]. DLD may be a candidate for clinical use because of its high reproducibility and fabrication in low-cost microfluidic devices.

Many microfluidic DLD devices with micropillars are produced using polydimethylsiloxane (PDMS), which has been used for microfluidic channel preparation in soft-lithography technologies because of its high spatial resolution for the transfer of nanopatterns (approximately 1 nm), optical transparency, and biocompatibility [24]. However, PDMS must be purified to medical grade for clinical use [25]. Conventional PDMS for non-medical purposes is not appropriate for medical applications, and it is especially difficult to use treated cells on PDMS-based chip devices for clinical treatments such as apheresis and implantation. Because PDMS for medical purposes is expensive, polypropylene (PP)-based chips have been used for cell treatments in medical applications because they are cost effective. Previous studies used elastomers and fabricated DLD devices via the hot-emboss method [17,18]. Our microfluidic DLD devices are made from PP-based resins. The devices are used for blood cell analytical devices [26,27] and their microstructures in the channels are prepared using the hot-emboss method or the injection molding method.

In this study, we used PP-based microfluidic DLD devices to separate bovine and human blood cells. The separation efficiency depended on the micropillar structures in the microfluidic channels. The D_c_ values obtained by experimental evaluations using immunobeads were nearly the same as the calculated values. The average diameters of the bovine red blood cells (RBCs) and white blood cells (WBCs, mainly lymphocytes and neutrophils) were 5 µm and 10 μm, respectively. We used microfluidic DLD devices with D_c_ = 8–10 μm and collected WBCs as large microparticles. Using DLD microfluidic chips with different calculated D_c_, we evaluated the differences in leucocyte collection in animal species and types of separated blood cells. We evaluated WBCs using histochemical staining. Finally, we discuss the potential clinical applications of our PP-based microfluidic DLD devices for diagnosis and treatment.

## 2. Materials and Methods

### 2.1. Materials

Immunobeads (Irvine Scientific, California, CA, USA), bovine blood (Japan Bio Serum Co. Ltd., Fukuyama, Japan), and heparin-treated human blood (Rockland Immunochemicals Inc., Pennsylvania, PA, USA) were purchased. Bovine blood cells and human blood cells were stained using the Wright–Giemsa stain kit (ScyTec Laboratories Inc., Utah, UT, USA) and Diff-Quik stain kit (Sysmex, Kobe, Japan), respectively. The running buffer was prepared using 0.5% (*w/v*) bovine serum albumin (BSA; Nacalai Tesque, Kyoto, Japan), and 2 mM EDTA (Dojindo, Kumamoto, Japan) in 10 mM PBS (Wako, Tokyo, Japan).

### 2.2. Device Fabrication

A schematic of the microfluidic DLD channel structure is shown in Figure 1b. This device has a micropillar array, two inlets, and two outlets. The silicon masters for the microfluidic DLD devices were fabricated at the Toyama Industrial Technology Research and Development Center using deep reactive ion etching (MUC-21 ASE-SRE, Sumitomo Precision Products Co., Ltd., Amagasaki, Japan) on a silicon wafer. PP-based tops and bottoms with microfluidic device micropillars were fabricated using an injection molding method and a silicon master mold (Richell Corporation, Toyama, Japan). The top and bottom were pressed together at 100 °C for 4 h, and the microfluidic channel was prepared by sealing the two units. The prepared microdevices were then placed in a polyacrylic manifold.

Figure 1c–e shows a photograph of the PP-based microfluidic DLD device, microscopic images of the inlets and outlets, and the pillar size and gaps of the micropillars of the device, respectively. G_h_ and G_v_ are the same in this device. The D_c_ values for the microfluidic DLD device are listed in Table 1. The channel height is the same as the height of the micropillars, which is approximately 40 μm (Figure 1f,g). The micropillar size and gap were proportionally changed to alter D_c_, whereas the array rotation angles were the same. Optical microscopy and scanning electron microscopy (SEM) images were recorded using Ts2R (Nikon, Tokyo, Japan) and JSM-6610LA (JEOL Ltd., Akishima, Japan), respectively. Three-dimensional images were reconstructed using VX-100 (Keyence, Osaka, Japan).

### 2.3. Methods

(1) Air pressure pumping system for microparticle separation

**Figure 2:**
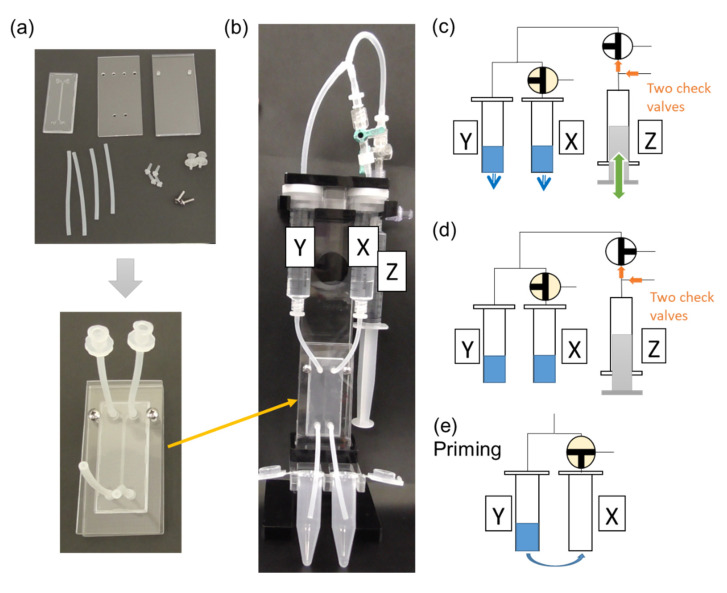
(**a**) Photographs of setting the microfluidic DLD device into a polyacrylic manifold. (**b**) Connection of the air pressure pumping system to the microfluidic DLD device. (**c**) A schematic representation of the mechanism to make fluids flow inside the microfluidic channel by increasing air pressure inside syringes X and Y. The two check valves work to increase the air pressure in syringes X and Y. (**d**) A schematic representation of the mechanism to stop fluid flow and restore atmospheric pressure inside syringes X and Y by turning the three-way stopcock. (**e**) A schematic representation of the mechanism of air removal in the microfluidic channel during the initial run.

The microfluidic channel was set in a polyacrylic manifold to connect the plastic syringes, as shown in Figure 2a. We developed an air pressure pumping system without an electric device, as shown in Figure 2b. Two 10 mL plastic syringes (Terumo, Tokyo, Japan) were used as the inlet reservoirs. The air pressure of the inlet syringes is the driving force for pushing the running buffer and sample suspension into the microfluidic DLD channel. After pushing and pulling the side syringe Z several times, as shown in Figure 2c, the air pressure inside syringes X and Y increases. Specifically, by pushing syringe Z, air is supplied to syringes X and Y; by pulling syringe Z, air is resupplied to syringe Z without decreasing the air pressure inside syringes X and Y via the two check valves; by alternating the pushing and the pulling, the air pressure inside syringes X and Y increases as the pushing time increases.

The flow rates were calculated from the fluid volumes of the outlet tubes for 5 min. The air pressure inside syringes X and Y and the flow rate of both outlets of Chip 1 were measured when running the buffer to the chip (Figure S1). The results are summarized in Figures S2 and S3. The flow rates of both outlets were proportional to the air pressure of the syringes. For the separation experiments, to maintain the air pressure, syringe Z was sometimes pushed by hand until it could not be pushed. The pressure in the experiments for immunobeads and blood cell separations was 200–260 kPa. As shown in Figure S2, the experimental results using a manometer to measure the air pressure inside syringes X and Y suggest that the flow rate decreases were approximately 12%, although a slight pressure change was observed. To facilitate the separation experiment, the maximum air pressure condition was set. The required air pressure changed to maintain the air pressure inside the syringe and the flow rate was small. Fluid flow can be stopped by turning the three-way stopcock, as shown in Figure 2d.

(2) Priming process

All devices, including the microfluidic DLD channel, were set on the vertical microfluidic chip holder, as shown in Figure 2b. To remove air inside the microfluidic channel during the initial priming process, the three-way stopcock (highlighted in yellow in Figure 2e) was turned to open syringe X, and the running buffer was only added to syringe Y. By pushing and pulling the side of syringe Z, the running buffer was transferred from syringe Y to syringe X. The three-way stopcock was then returned to the initial position (Figure 2c). The running buffer in both syringes X and Y flowed to the outlets, and the air moved away from the microfluidic DLD channel.

(3) Immunobeads and blood cell separation

After the priming process, the sample suspensions were added to the right syringe, and air pressure was applied to the sample and running buffer to create laminar flows. When 1–3 mL of fluid was taken from the outlet tubes, the air pressure was released, and the separation process was completed. Immunobeads were diluted 10 times with the running buffer for the separation experiments. The dilution ratio was 4 for both bovine and human blood. Blood cells were counted with a microscope and a hematocytometer. The concentration of bovine blood and human blood was 3 million cells/µL and 0.9 million cells/µL, respectively. When the diluted bovine and human blood was inputted, the flow sample volume of the blood was less than 1 mL, because of clogging of the RBCs at the inlet of the microfluidic channel. The clogging phenomenon was only observed when we used purchased heparin-treated blood and not when fresh human blood was used (see discussion). In our previous study, EDTA-treated fresh human blood was only diluted with PBS twice for a microfluidic DLD chip made of a different material and the same microfluidic channel dimensions of Chip 1 [28].

An aliquot of the fluid in the right outlet tube was analyzed using a microscope equipped with a CCD camera (HAS-10, Detect Co. Ltd., Osaka, Japan). For WBCs, the fluid in the left outlet tube was centrifuged at 1000 rpm for 5 min (CF16RXII, Hitachi-Koki, Ibaraki, Japan) to precipitate the cells at the bottom of the tube, and the fluid with the precipitate was recovered and analyzed. The diameters of the separated particles were analyzed using NIH Image J software (National Institutes of Health, Bethesda, MD, USA).

(4) Blood cells staining

Bovine and human blood cells were stained with Wright–Giemsa and Diff-Quik staining kits to observe the internal structure of the WBCs. The stained cell images were recorded using an optical microscope (TS100, Nikon) equipped with a color CCD camera (DS-Fi2, Nikon).

(5) Real-time imaging of the beads and blood cell separation

A microscopic system for microfluidic channel observation from the side was prepared, as shown in Figure S4a. Figure S4b shows the optical alignment of the system using a 20× magnification lens (Nikon) and CCD camera (HAS, Detect). Particle sorting images were recorded around the outlet channels (Figure S4c). The frame rates of the movies were 250 fps. The average diameters of polystyrene beads were 12 μm, 10 μm, and 8 μm (Duke Standards, Thermo Fisher Scientific, Waltham, MA, USA). Fifty microliters of the bead suspension was diluted with 1 mL of running buffer.

## 3. Results

### 3.1. Immunobead Separation

**Figure 3:**
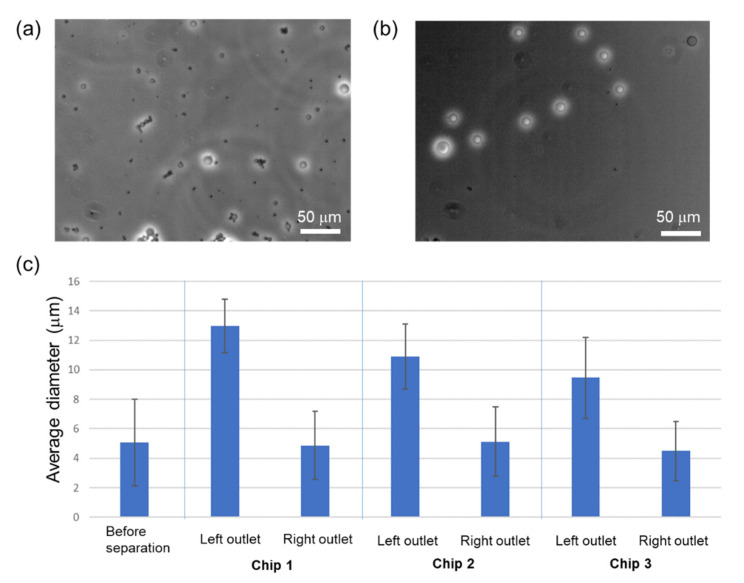
Microscopic images of immunobeads (**a**) before separation and (**b**) after separation in the left outlet using Chip 1. (**c**) Average diameter of immunobeads in the left and right outlets. The error bars show the standard deviation. The sample number in each group is 20–100.

**Figure 4:**
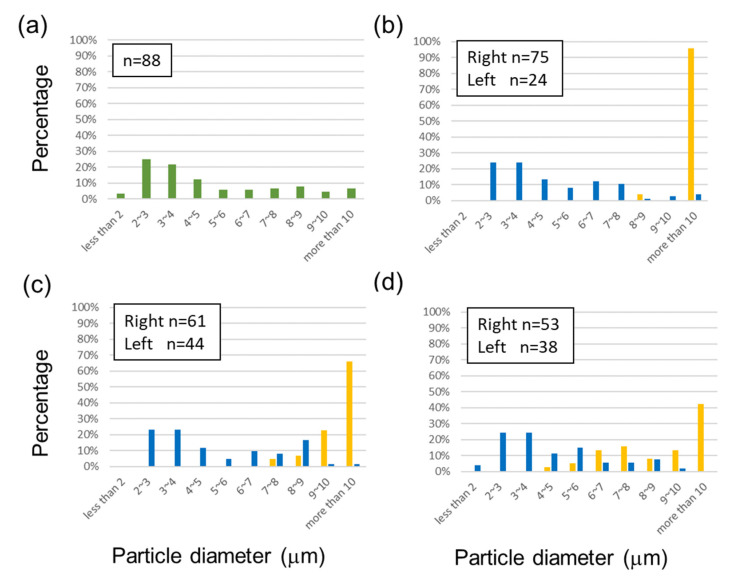
Histogram of immunobead diameter distribution (**a**) before separation and after separation using (**b**) Chip 1, (**c**) Chip 2, and (**d**) Chip 3. Blue and yellow bar graphs show the diameter distributions in the right and left outlets, respectively. A droplet of 5–10 μL was applied to the slide glass from 0.5–1.0 mL of the outlet sample, the droplet was covered with a thin glass, and we observed 5–10 positions (n = 20–100). The percentage of counted immunobeads was 0.1–1% of the total sorted beads.

Figure 3a,b shows the optical microscopy images of the spherical immunobeads before sorting and at the left outlet of Chip 1, respectively. Larger particles were separated in the left outlet. Figure 3c shows the average diameter of immunobeads before and after DLD sorting. The average diameter correlated with the D_c_ value of each chip. Histograms of the bead diameter distribution before and after sorting using Chips 1, 2, and 3 are shown in Figure 4a–d, respectively. When all of the chips were used, the average diameters in the left outlet were greater than those in the right outlet, suggesting that spherical particles with larger diameters were sorted based on the DLD mechanism.

### 3.2. Bovine Blood Cell Separation

**Figure 5:**
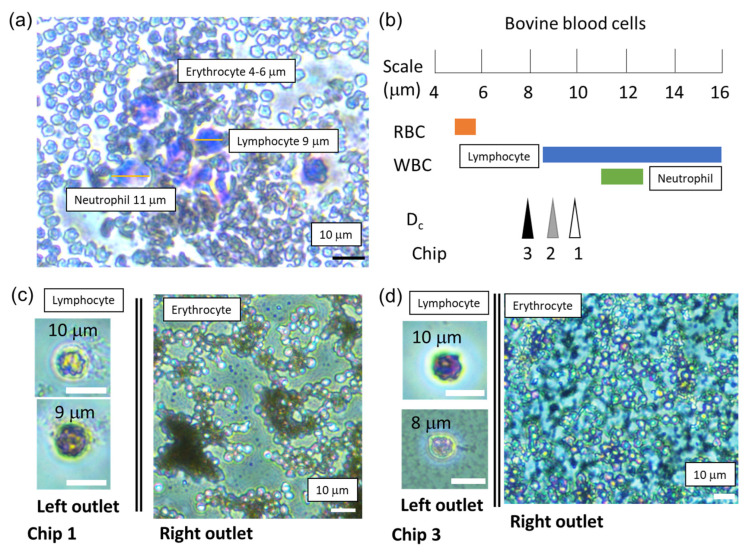
(**a**) A microscopic image of bovine blood cells before sorting. (**b**) Comparison of the diameters of bovine blood cells [29,30,31,32] and D_c_ values of the microfluidic DLD devices. Microscopic images of bovine blood cells collected at the left and right outlets using (**c**) Chip 1 and (**d**) Chip 3, respectively. The scale bars are 10 μm.

Bovine blood cell separation experiments were conducted using Chips 1, 2, and 3. Figure 5a shows a microscopic image before the separation of bovine blood cells (stained with the Wright–Giemsa reagent). Erythrocytes (RBCs), lymphocytes (WBCs), and neutrophils (WBCs) were observed. Figure 5 compares the diameter of these blood cells and D_c_ of the microfluidic DLD device. Lymphocytes and neutrophils are the majority of bovine WBCs [29,30,31,32,33]. Figure 5c,d shows microscopic images of the sorted blood cells using Chips 1 and 3, respectively. Lymphocytes were observed in the left outlet of all of the three chips, whereas RBCs were not observed. However, only erythrocytes were recognized at the right outlet of these chips. The mean and maximum diameter of bovine erythrocytes is approximately 5.1 µm and 5.5 μm, respectively, which is 2 μm less than the D_c_ of Chip 3 [34]. Therefore, using the microfluidic DLD device, RBCs and WBCs were efficiently separated, because the difference in average diameters between that of the RBCs and the sorted lymphocytes was approximately 3 μm (Figure 5b).

### 3.3. Human Blood Cell Separation

**Figure 6:**
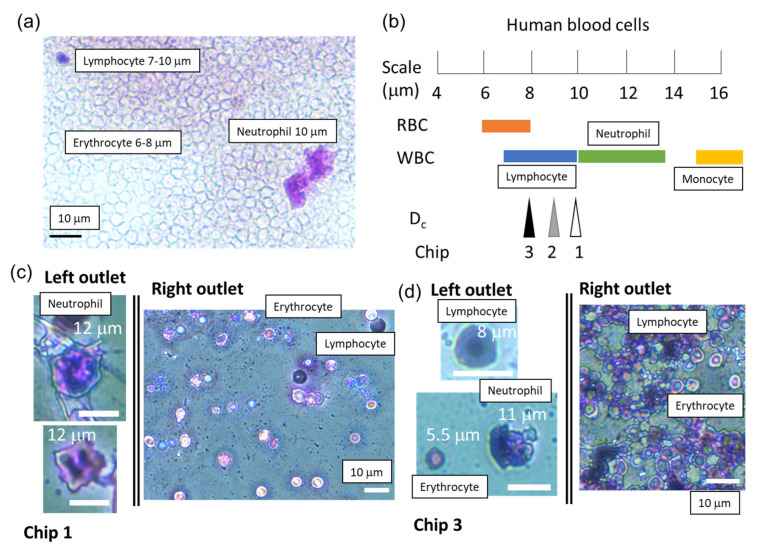
(**a**) A microscopic image of human blood cells before sorting. (**b**) Comparison of the diameters of human blood cells and D_c_ values of the microfluidic DLD devices [35]. Microscopic images of collected human blood cells from left and right outlets using (**c**) Chip 1 and (**d**) Chip 3, respectively. The scale bars are 10 μm.

The distribution of human blood cell size is different from that of bovine blood cells, and the separation results are influenced by the cell size distribution. Figure 6a shows the microscopic image of human blood cells before the sorting experiment. Erythrocytes, lymphocytes, and neutrophils were also detected in this image. As shown in Figure 6b, the distribution of the diameter of the human RBC (6–8 μm) overlaps with that of the human lymphocytes (7–10 μm) [35]. The diameters of neutrophils and monocytes are usually >10 μm [35]. Figure 6c,d shows the human blood cells that were collected using Chips 1 and 3, respectively. As shown in Figure 6c, neutrophils were sorted in the left outlet and erythrocytes and lymphocytes in the right. As shown in Figure 6d, lymphocytes and neutrophils (WBC) sorted to the left outlet; however, some erythrocytes (approximately 10 cells/μL) were also observed, because the D_c_ value was approximately that of the human RBC (Figure 6b). Immunostaining is a superior method to histochemical staining for clear imaging of blood cell types. As a result, we are developing a recognition method by combining sorted blood cells and staining methods with microfluidic DLD devices and automated technologies [17,18].

### 3.4. Real-Time Imaging of the Beads and Blood Cell Separation

**Figure 7:**
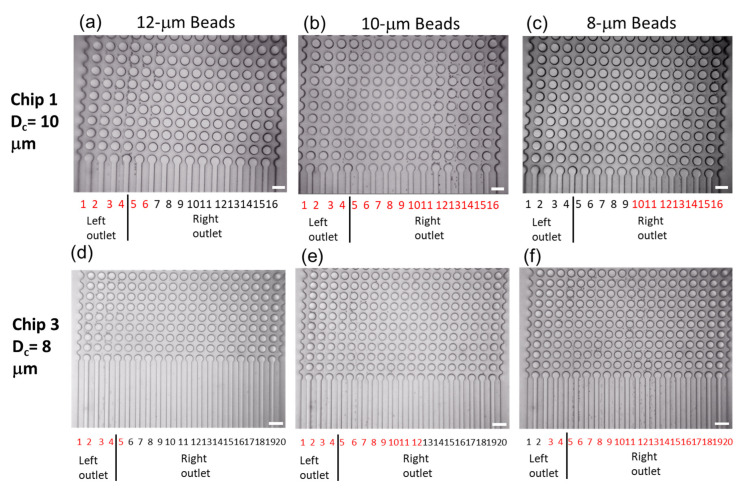
Images of polystyrene bead separations near outlet channels for 0.1 s (25 frames). (**a**) 12-µm beads, (**b**) 10-µm beads, or (**c**) 8-µm beads separated using Chip 1 in which Channels 1–4 are connected to the left outlet and Channels 5–16 are connected to the right outlet. (**d**) 12-μm beads, (**e**) 10-µm beads, or (**f**) 8-μm beads separated using Chip 3 in which Channels 1–4 are connected to the left outlet and Channels 5–20 are connected to the right outlet. The outlet channel numbers in red indicate sorted particles that were observed in the outlet channel. The scale bars are 100 μm.

To determine the relationship between the sorted particle diameter and the calculated D_c_, we recorded sorting images of polystyrene beads with 12-µm, 10-µm, and 8-µm diameters, as shown in Figure 7. As the bead diameter decreases, the outlet channels with the sorted beads shifted to the right. When Chip 1 was used, 12-µm and 10-µm beads flowed into the left outlet, while 8-µm beads were only sorted into the right outlet. Using Chip 3, some of the 8-µm beads were observed in the left outlet. The observed D_c_ values were similar to the values calculated in our experimental condition.

**Figure 8:**
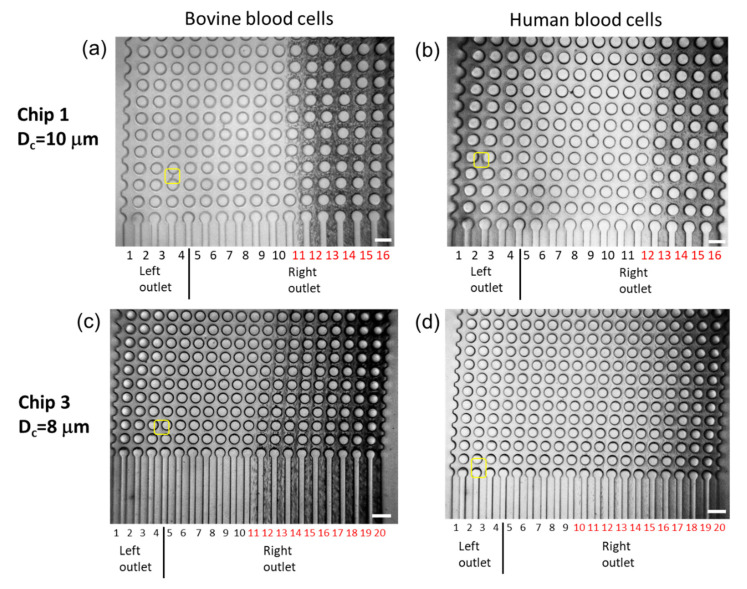
Images of bovine and human blood cell separations around outlet channels (one frame). (**a**) Bovine blood cells or (**b**) human blood cells separated using Chip 1 in which Channels 1–4 are connected to the left outlet and Channels 5–16 are connected to the right outlet. (**c**) Bovine blood cells or (**d**) human blood cells separated using Chip 3 in which Channels 1–4 are connected to the left outlet and Channels 5–20 are connected to the right outlet. The outlet channel number in red represents the observation of RBCs in the outlet channel. The particles in yellow squares are sorted into the left outlet. The scale bars are 100 µm.

Figure 8 shows bovine and human blood cells around the outlet channels during the sorting experiments. The sorted particles flowing to the left outlet, as indicated by the yellow squares in Figure 8, are WBCs. The percentage of sorted WBCs before the sorting was approximately 0.1%. Bovine and human RBCs were considered as 5-µm and 6–8-µm microparticles, and they sorted into the right outlet of both Chip 1 and Chip 3. Alternatively, as shown in Figure 6d, RBCs were occasionally observed in the left outlet of Chip 3, indicating that some RBCs may have migrated because of the greater deviation in the sorting property, as shown in Figure 4d. To prevent RBC migration to the left outlet, the calculated D_c_ of the DLD microfluidic channel should be substantially greater than the diameter of the RBCs.

## 4. Discussion

In the separation of blood cells using DLD, the diameter distribution of erythrocytes and lymphocytes is an important factor to consider for preventing the collection of erythrocytes at the left outlet. All chips were appropriate for the separation of bovine RBCs and WBCs. However, Chip 1 was more suitable for human blood cell separation. The separation properties of the blood cell diameter in this study were similar to those in previous studies that used human blood cells. When a 1 mL sample of blood cells diluted five times (Ht = 3–5) was treated in the separation experiments using these chips, the number of WBCs detected at the left outlet was approximately 10 in 10 μL. To overcome the low WBC concentration, we developed a microfluidic DLD channel that could concentrate the sorted particles inside the device, and the concentrated cells could be analyzed using flow cytometry. To show the effectiveness of this device for WBC collection in molecular biological analyses, diagnosis, and cell-based treatments, the sample preparation and experimental systems, such as the concentration of WBCs and selective cell labeling, must be optimized.

Fluid mechanical insights are important for high-speed imaging that is faster than the video rate [4,6]. These imaging experiments allow us to evaluate the blood cell collision effects on RBC and WBC separation [22], variances in D_c_ values based on the Reynolds number (Re) of the fluid [9], and the effects of the wall surface on fluid mechanical environments. In the immunobead separation, D_c_ calculated using Equation (1) and the observed D_c_ values were the same, as shown in Figure 7. The relationship of the flow rate of the outlets (~0.2 mL/min at the maximum) in this experiment and the references were verified [6,23]. The Re of this fluid flow is less than 10, and the effective critical diameter (D_c_) in the experiments is similar to the calculated values (at most a 10% decrease) according to the correlation between Re and D_c_ [23]. Additionally, computational fluid dynamics simulation results suggest that the D_c_/Gap decrease is approximately 3%, from Re = 1 to Re = 10 [9]. Throughout this study, the experimental D_c_ was similar to the calculated D_c_. A previous study suggested that as the hematocrit increases, the theoretical D_c_ decreases [22]. The hematocrit dependency and blood cell deformation in the microfluidic DLD channel may affect D_c_, and these effects are evaluated using high-speed imaging.

Regarding the higher deviation when the D_c_ of the DLD microfluidic chip decreases, as the microfluidic channel dimension decreases, the interaction between the particles and walls enhances. Particle collision effects may then increase this deviation as D_c_ decreases [22]. The enhanced particle collision effects and/or particle–wall interactions may alter the sorting property of microparticles in the DLD microfluidic channel, which may induce more deviation. The relationship between the particle–particle and particle–wall interactions and channel dimension will be discussed in another study.

Microfluidic DLD devices can be used for the diagnosis of bovine blood diseases, such as bovine leukemia virus (BLV) infections, where the WBC count can be used as a marker [36,37]. Collective recovery of WBCs using this device may facilitate microscopic analyses by omitting RBCs from the livestock chamber [36,37]. The diagnosis of human blood diseases using DLD devices has been previously studied [17,18,19]. DLD devices can potentially be used in plasma exchange applications for blood purification, such as apheresis. Clinically, we must optimize the structure of the device to suit practical applications, such as the scale-up and concentration of sorted cells. We observed clogging of the blood cells in the right inlet while using heparin-treated whole blood that is commercially available. This clogging may be reduced by the addition of reagents that reduce the blood coagulation system and/or activate the fibrinolysis system. As mentioned above, microfluidic DLD devices made of PP-based resins can be used for diagnostic and clinical applications [17,19,28].

## 5. Conclusions

We have developed a microfluidic DLD device using PP-based resin that works similarly to those prepared using PDMS. Air pressure-driven systems can be used in cattle houses and fields where using an electric power source is difficult. In our experimental condition (Re < 10), a maximum flow rate of 0.2 mL/min did not influence the calculated D_c_. We suggest that a chip with a calculated D_c_ should be applied to the blood cells of each animal species using this electricity-free setup. Bovine RBCs and WBCs were separated using microfluidic DLD devices with D_c_ = 8–10 μm, and this system can be applied to WBC collection and the diagnosis of BLV infections in cattle houses. Separation of human RBCs and WBCs using these devices was more difficult than separating bovine blood cells because of the similarity in diameters between human erythrocytes and lymphocytes. Because of the biosafety of PP-based resins, microfluidic DLD devices may be adopted for diagnosis and clinical treatments such as blood cell collection and plasma exchange treatment.

## Figures and Tables

**Table 1:** Summary of the microstructures of the deterministic lateral displacement (DLD) microdevices in this study.

Chip No.	D_c_ (μm) ^1^	Gap (*g*) (μm)	tan(θ)	Flow Rate Left Outlet (mL/min)	Flow Rate Right Outlet (mL/min)
1	10.0	30	0.05	0.09 ± 0.005 ^2^	0.17 ± 0.014 ^2^
2	9.0	27	0.05	0.09 ± 0.010 ^2^	0.21 ± 0.008 ^2^
3	8.0	24	0.05	0.06 ± 0.026 ^2^	0.11 ± 0.035 ^2^

^1^ D_c_ was calculated using Equation (1). ^2^ Averages ± standard deviation (N = 3 or 4).

## Data Availability

Not applicable.

## Supplementary Materials

The following supporting information can be downloaded at: https://www.mdpi.com/article/10.3390/mi14020238/s1. Figure S1: an illustration of the air channel to set a digital manometer for air pressure detection inside syringes X and Y; Figure S2: air pressure changes inside syringes X and Y of (a) Chip 1, (b) Chip 2, and (c) Chip 3; Figure S3: correlation between the pressure and outlet flow rates of (a) Chip 1, (b) Chip 2, and (c) Chip 3; Figure S4: (a) a photograph of the observation setup from the side. (b) A schematic presentation of optical alignment. (c) A microscopic image of the outlet entrance of Chip 1.
