# Supplementary material for: Blood Cell Separation Using Polypropylene-Based Microfluidic Devices Based on Deterministic Lateral Displacement
Source: Micromachines (Basel). 2023 Jan 17;14(2):238. doi: 10.3390/mi14020238 (PMC9960998; doi:10.3390/mi14020238)
Supplement: Supplementary file 1 [file micromachines-14-00238-s001.zip › micromachines-2092851-supplementary.pdf]

Supplementary Materials

Blood cell separation using polypropylene-based microfluidic devices based on deterministic lateral displacement

Koji Matsuura [1, *] and Koji Takata [2]

1 Department of Biosciences, Faculty of Life Science, Okayama University of Science; kmatsuura@ous.ac.jp

2 Life Materials Development Section, Human Life Technology Research Institute, Toyama Industrial Technology Research and Development Center; takata@itc.pref.toyama.jp

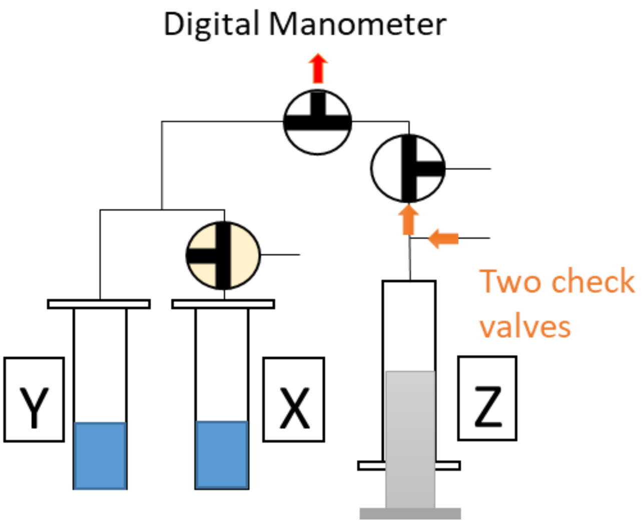

Figure S1. An illustration of the air channel to set a digital manometer for air pressure detection inside syringes X and Y.

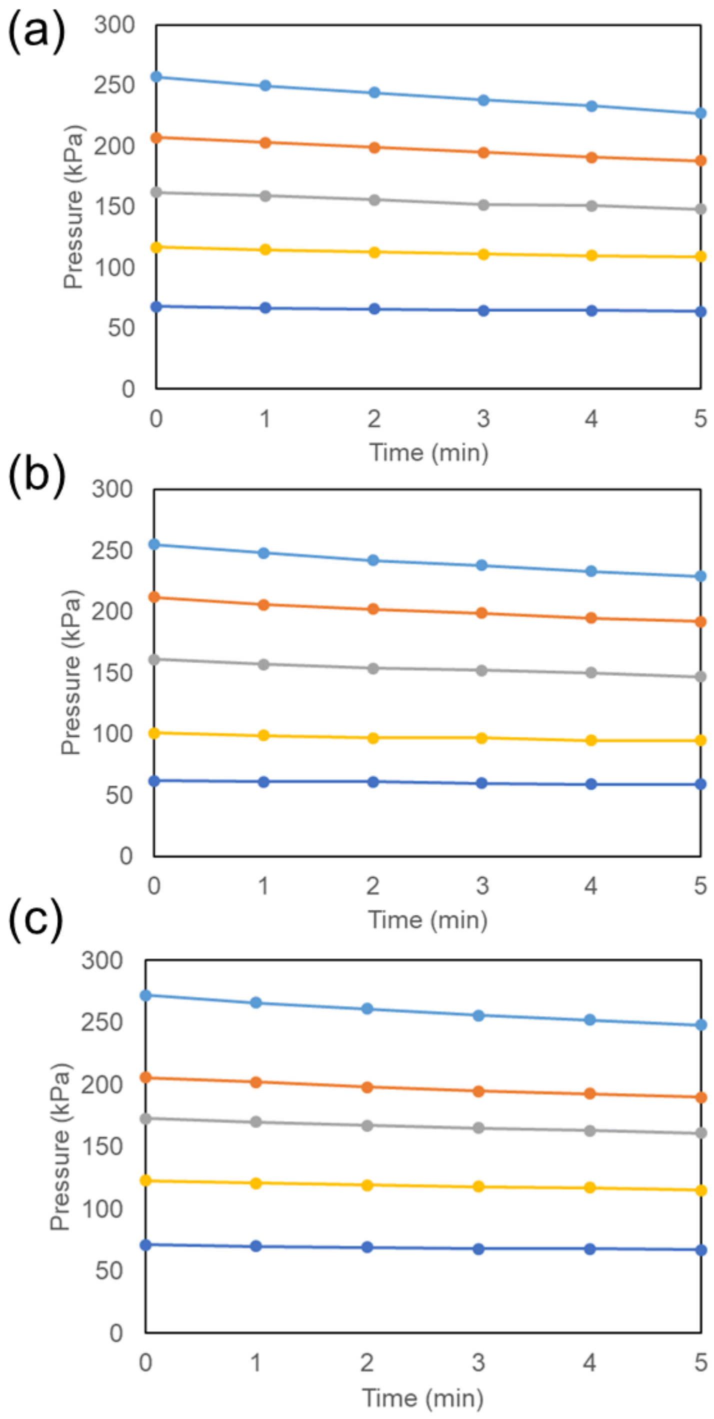

Figure S2. Air pressure changes inside syringes X and Y of **(a)** Chip 1, **(b)** Chip 2, and **(c)** Chip 3. The pressure drop of all of the chips over 5 min were similar.

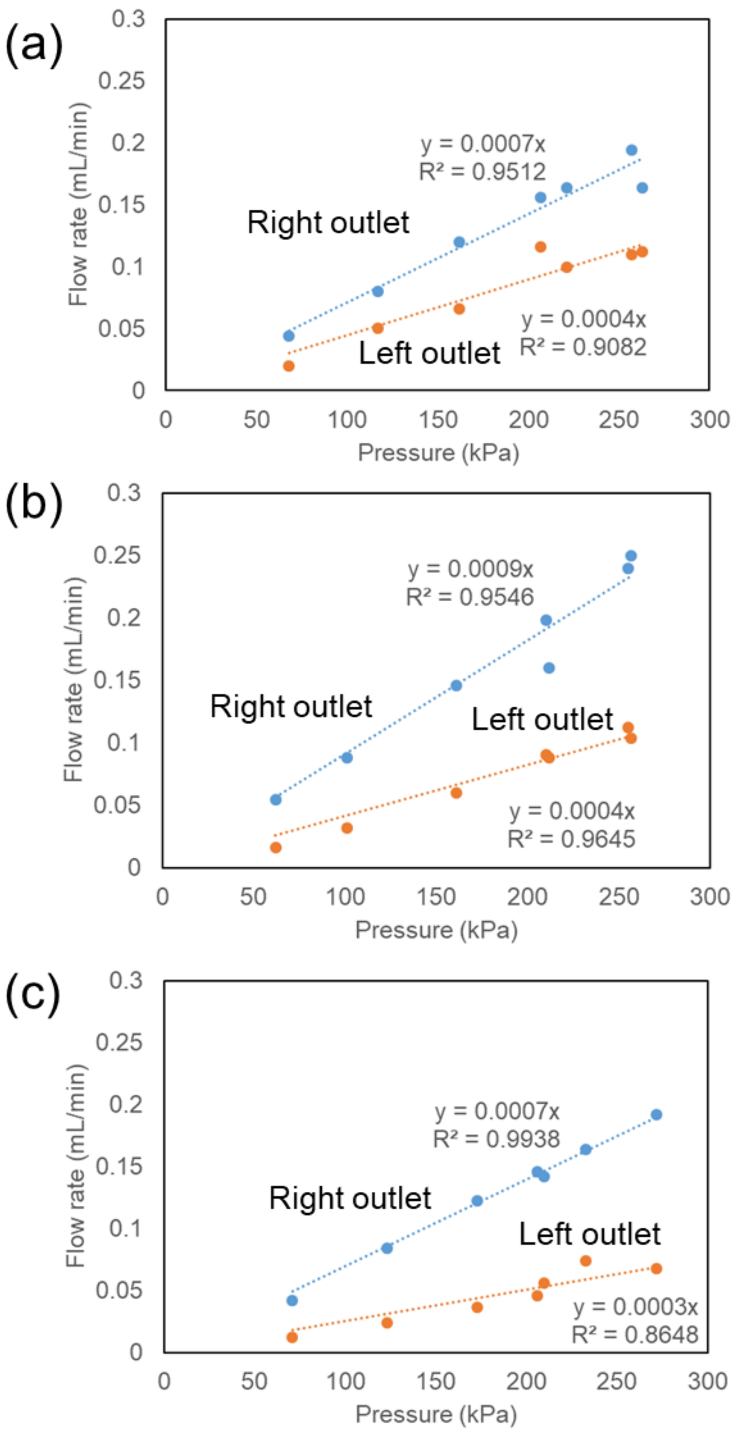

Figure S3. Correlation between the pressure and outlet flow rates of **(a)** Chip 1, **(b)** Chip 2, and **(c)** Chip 3. The flow rate was proportional to the air pressure inside syringes X and Y.

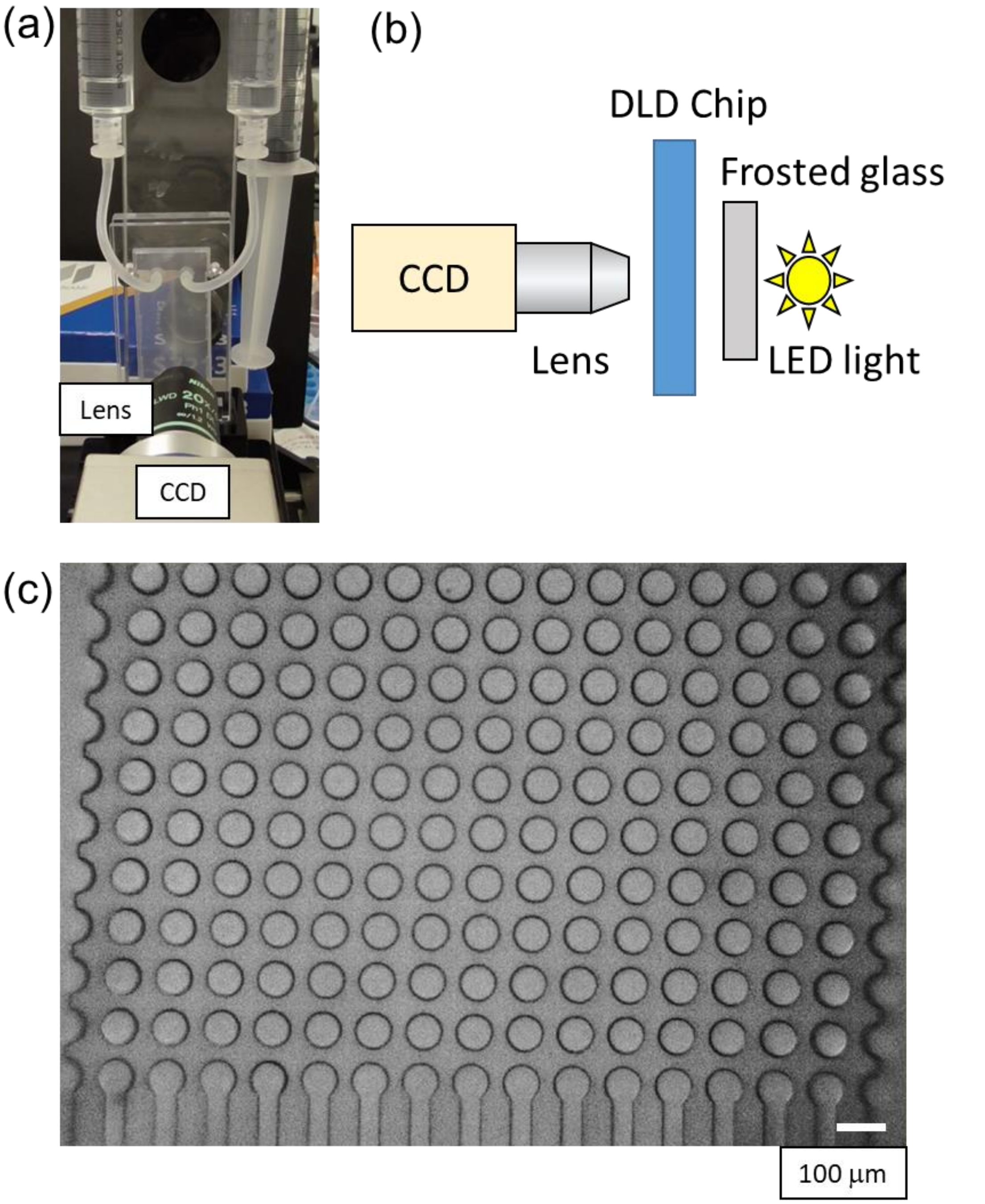

Figure S4. **(a)** A photograph of the observation setup from the side. **(b)** A schematic presentation of optical alignment. **(c)** A microscopic image of the outlet entrance of Chip 1.
